# Changes in Climate Vulnerability and Projected Water Stress of The Gambia's Food Supply Between 1988 and 2018: Trading With Trade-Offs

**DOI:** 10.3389/fpubh.2022.786071

**Published:** 2022-05-25

**Authors:** Genevieve Hadida, Zakari Ali, Thomas Kastner, Tony W. Carr, Andrew M. Prentice, Rosemary Green, Pauline Scheelbeek

**Affiliations:** ^1^Department of Population Health, London School of Hygiene & Tropical Medicine, London, United Kingdom; ^2^Nutrition Theme, MRC Unit The Gambia at the London School of Hygiene and Tropical Medicine, Banjul, Gambia; ^3^Senckenberg Biodiversity and Climate Research Centre Senckenberg, Frankfurt am Main, Germany; ^4^Centre on Climate Change and Planetary Health, London School of Hygiene & Tropical Medicine, London, United Kingdom

**Keywords:** climate change, food security, trade, food system resilience, adaptation, water stress, environmental change, climate vulnerability

## Abstract

**Background:**

The coexistence of under- and overnutrition is of increasing public health concern in The Gambia. Fruits, vegetables and pulses are essential to healthy and sustainable diets, preventing micronutrient deficiencies and non-communicable diseases, while cereals significantly contribute to energy intake. However, environmental changes are predicted to intensify, reducing future yields of these crops if agricultural productivity and resilience are not improved. The Gambia is highly climate-vulnerable and import-dependent, but the extent of its reliance on other climate-vulnerable countries for its supply of nutritionally important crops is currently unknown.

**Methods:**

We used United Nations Food and Agriculture Organization data, with novel origin-tracing algorithms applied, to analyse The Gambia's supply of cereals, fruits, vegetables and pulses between 1988 and 2018. The climate vulnerability of countries was assessed using Notre Dame Global Adaptation Initiative (ND-GAIN) index scores, and projected water stress (2040) assessed using World Resources Institute (WRI) scores. Multilevel generalized linear mixed-effects models were used to identify changes in the overall climate vulnerability and projected water stress of supply.

**Results:**

Between 1988 and 2018, The Gambia's supply of cereals, fruits, vegetables and pulses diversified, with the proportion domestically produced falling (Cereals: 61.4%–27.7%; Fruits: 93.0%–55.7%; Vegetables: 24.6%–16.3%; Pulses: 100.0%–76.0%). The weighted-average ND-GAIN scores improved (indicating less climate vulnerability) for supply of all crops except cereals, but the weighted-average WRI score for supply deteriorated (indicating increased projected water stress) for all crops except vegetables. When just considering imports, weighted-average ND-GAIN scores deteriorated for fruits and cereals while showing no significant change for other food groups, and the WRI score deteriorated for cereals only.

**Conclusions:**

Despite some notable improvements in the environmental vulnerability of The Gambia's supply of nutritionally important crops (particularly vegetables), considerable, and in some cases increasing, proportions of their supply are produced in countries that are vulnerable to climate change and future water stress. This may have implications for the availability, affordability, and hence consumption of these crops in The Gambia, ultimately exacerbating existing nutritional challenges. Exploring the options to strengthen supply resilience—such as altering trade patterns, agricultural techniques and diets—should be prioritized.

## Introduction

Environmental change is adding pressure to an already fragile global food system (1). Climate change, water shortage, heat stress and biodiversity loss are predicted to intensify in the coming decades (2) with substantial implications for food production (1). Without action, research shows that projected environmental changes will reduce yields of nutritionally important crops such as fruits, vegetables and pulses, and staples such as rice and wheat (3–5). With fruits, vegetables and pulses contributing to a diverse diet, the prevention of micronutrient deficiencies, obesity, and non-communicable diseases (6), and cereals a staple energy source in diets worldwide (7), environmental change may therefore compound existing nutritional challenges experienced by countries.

The Gambia is a semiarid country (8) which, as well as facing an increasing coexistence of under- and overnutrition (9, 10), is considered highly vulnerable to climate change. This classification stems from its high climate variability and reliance on rain-fed agriculture, alongside its limited economic and infrastructural capacity to respond to such stressors (11). Despite being classified among the least water-stressed countries in the world (as, in the general absence of irrigation practices, total water withdrawal accounts for little of the total renewable water resources), the main risk associated with crop production is the increasing frequency of extreme weather events such as floods and droughts (12). Yearly variations in food production follow rainfall trends, and the short rainy season between June and September limits food production to only one cropping season. As a result, there is currently a strong reliance on imports, and hence the capacity of other countries, to supplement The Gambia's increasing food demand (8).

Since the agricultural impacts of environmental change on countries varies, international trade has been proposed as a potential food system adaptation to strengthen resilience and safeguard food security (13–16). Resilience is a concept increasingly being used to address food system challenges. There is some variation in how the concept is defined, but generally it refers to the “capacity over time of a food system and its units at multiple levels, to provide sufficient, appropriate and accessible food to all, in the face of various and even unforeseen disturbances” (17). Given the complexity of food systems it is difficult to measure resilience, but here we focus on two factors that relate to it: the climate vulnerability and projected water stress of countries that are a source of imported crops for The Gambia.

Current trading patterns are driven by economic incentives, established supply-chain relationships, and consumer demand, and not directly by relative environmental impacts. With this, there is some suggestion that these are not typically following sustainable patterns (i.e., exports by climate-stable countries to climate-vulnerable ones), rather the opposite. A recent analysis of the United Kingdom's fruit and vegetable imports has shown that over time, the proportion coming from climate-vulnerable countries, and countries projected to be water-stressed by 2040, has significantly increased (18). While trade patterns are inherently complex and likely to change in response to environmental change themselves (16), this provides some indication that supplies may become more vulnerable to environmental change, even in countries where this is expected to have less of a direct impact, such as the United Kingdom.

For climate-vulnerable, import-dependent countries that are prone to drought, such as The Gambia, the need for trade flow efficiency is even greater because, without major increases in agricultural productivity, there is a risk that their domestic resource will become increasingly limited. While the complexity of food systems and trade make it difficult to predict, there is a risk that significant reliance on other climate-vulnerable countries may add to the environmental vulnerability of The Gambia's food system, potentially reducing the availability and affordability of crops (19, 20) as supplies could decrease. This could carry negative implications for food security and overall population health (1, 20).

Given The Gambia's reliance on food imports (8), which may increase due to environmental change and challenges in food production domestically, choosing trade partners that are more climate and water-stable could play a part in the country's climate adaptation strategy. While a more holistic assessment of potential policy options would ultimately be necessary, knowledge of the degree of dependency on crop imports from other vulnerable countries is a relevant starting point for Gambian policymakers. We therefore combine several databases to analyse current supply and trade flows of cereals, fruits, vegetables and pulses to The Gambia, in terms of the overall climate vulnerability and projected water stress of their trade partners.

## Materials and Methods

This secondary data analysis combines several open-source datasets to conduct cross-sectional and longitudinal analyses of The Gambia's international trade of cereals, fruits, vegetables and pulses, between 1988 and 2018.

### The Gambia's Trade of Fruit, Vegetables, and Cereals

To estimate historical trends in The Gambia's per capita supply of cereals, fruits, vegetables and pulses, we used the open-source FAOSTAT food balance sheets between 1987 and 2018. To analyse The Gambia's international trade patterns we relied on a modified version of the FAOSTAT bilateral trade flows and production data for the years 1987–2019. This modified version establishes clear links between trade in primary crops and processed crop products, as captured in FAOSTAT, between the countries growing the crops and the countries of apparent consumption of the respective crop, eliminating transit countries that distort official trade statistics (21). The approach utilizes matrix algebra and is based on the assumption that domestic production and imports of a given crop proportionally contribute to domestic consumption and to exports. Further details are provided by Kastner et al. (21). Applying this method yielded data on the origin of The Gambia's supply for 64 primary crops and 204 countries. Crops were subsequently categorized into food groups, allowing a new trade matrix to be constructed for cereals (consisting of 13 individual crops), fruits (22), vegetables (21) and pulses (6), respectively (Supplementary Table 1). New variables for these four food groups were created, so that analysis could take place by food group level.

Using the data described above for proportional imports from trade partners, the import dependency ratio (IDR) of each food group was calculated by dividing the sum of imports by the sum of imports plus domestic production, minus exports, and multiplying by 100, following **Equation 1** (22). The equation was calculated in terms of crop quantities, and therefore the units of all values are metric tons. The complement of the IDR (e.g., 100–IDR) represents the part of available supply from The Gambia's own production. The calculated IDR and its complement were applied to the total quantity of supply data, which was taken directly from the FAOSTAT food balance sheets (considered the most reliable numbers for this).


(1) $$\begin{array}{l} IDR=\\ \;\frac{Imports\;(MT)}{Imports\left(MT\right)+\;Domestic\;Production\;\left(MT\right)-\;Exports\;(MT)}\;\times \;100 \end{array}$$

### Mapping Environmental Vulnerability of Trade Partners

Additional information regarding a country's environmental vulnerability was added to each country in the trade matrix (including The Gambia itself). To analyse the overall climate vulnerability of countries supplying cereals, fruits, vegetables and pulses, the Notre Dame Global Adaptation Initiative (ND-GAIN) country index scores were used. This is a composite measure of a country's exposure, sensitivity and capacity to adapt to climate change (23), and as such is considered the only thorough effort to quantify resilience at a national-level (24). While the index does not include indicators specific to fruits, vegetables and pulses (only overall agricultural capacity and projected cereal yields) (23), this index is considered a particularly comprehensive proxy for overall risk because of its inclusion of adaptive capacity. Adaptive capacity is often omitted in other indices, including several indices focusing on agriculture specifically, but has been shown to be a significant factor in the pressure climate change presents to countries (25). Scores are assigned at a country-level and therefore do not capture intra-country heterogeneity and localized risk; however, the index was selected because of its transparent methodology (23) and development in consultation with a wide range of academics, practitioners and private sector actors (26).

The latest ND-GAIN scores (2019) were projected onto The Gambia's supply from 1987 to 2019. Using these reflects the current situation, and—given that observed trends are overwhelmingly linear—using the latest available year is closest to what future situations may entail if supply follows these trends. Further, using the same scores (2019) for each year of analysis means that any change in the climate vulnerability of supply and imports over time can be solely attributed to changing trade partners. Countries of production and consumption were assigned a score. This score theoretically falls between 0 (most climate-vulnerable) and 100 (least climate-vulnerable), but currently country specific scores range from 28.3 to 76.8. We calculated quintiles of this range to categorize differing degrees of relative climate vulnerability ranging from 1 (most vulnerable) to 5 (least vulnerable).

To analyse likely future water scarcity, projected water-stress scores for 2040 [under a business-as-usual scenario constructed by the World Resources Institute (WRI)] were assigned to countries of production and consumption (27). Water stress measures total annual water withdrawals (municipal, industrial, and agricultural) as a percentage of the total annual available blue water. Countries are assigned a score between 0 and 5, where higher values correspond to greater competition among water users relative to available surface water resources. The established categories are: [0–1] <10%: low stress, [1–2] 10−20%: low-to-medium stress, [2–3] 20–40%: medium-to-high stress, [3–4] 40–80%: high stress and [4–5] >80%: extremely high stress (27).

Weighted average scores (*AS*) for climate vulnerability (ND-GAIN) and projected water stress (WRI)—where weights were assigned proportional to a country's contribution to 1. supply (domestic production plus imports minus exports) and 2. imports (supply with the contribution from domestic production removed)—were calculated for supply and imports of all crop groups, each year between 1987 and 2019, following **Equations 2a,b**.


(2a) $$\begin{array}{r} S_{x,p,c}\;=\;S_{p}\;\times \;T_{x,p,c} \end{array}$$

(2b) $$AS_{x,c}\;=\;\frac{\sum_{p=1}^{n}S_{x,p,c}}{\sum_{p=1}^{n}T_{x,p,c}}$$

Where *S* is the score (ND-GAIN or WRI) associated with the country of production *p*, *T* is the amount (MT) of traded crop *x*, *c* is the consuming country (The Gambia), and *n* is the number of supplying countries.

### Statistical Analysis

Three-year rolling averages were calculated for both proportions and weighted average scores, and we present data in 10-year intervals: 1988 (1987–1989); 1998 (1997–1999); 2008 (2007–2009) and 2018 (2017–2019).

Multilevel generalized linear mixed-effects models (GLMM) identified the relationship between the weighted average scores of supply and time (years), and the relationship between the weighted average scores of imports (supply with the contribution from domestic production removed) and time. Generalized models were used to account for non-linear trends within the data. While other models (including those for longitudinal data and recurrent trends) were explored, these did not show a better model fit than the GLMM.

Data were analyzed using STATA SE [version 17.0] and Microsoft Excel [2019]. ARCGIS version 10.8.1 (28) was used to map imports which contributed ≥1% of The Gambian supply of cereals, fruits, vegetables and pulses. The maps provided a visual tool linking the trade data to both climate-vulnerability and water-stress of countries.

### Missing Data

The raw trade data contained some negative values, probably the result of re-exports (goods imported into a country and then exported again). These were set to 0 and accounted for <0.1% of the data analyzed.

Not all trade partners have corresponding ND-GAIN and/or WRI scores, but this did not affect any of The Gambia's major suppliers (those contributing ≥1% supply). The proportions of supply with unknown ND-GAIN/WRI scores are included in analysis and presented in bar charts.

## Results

### Supply of Cereals, Fruits, Vegetables and Pulses (1988–2018)

**Figure 1:**
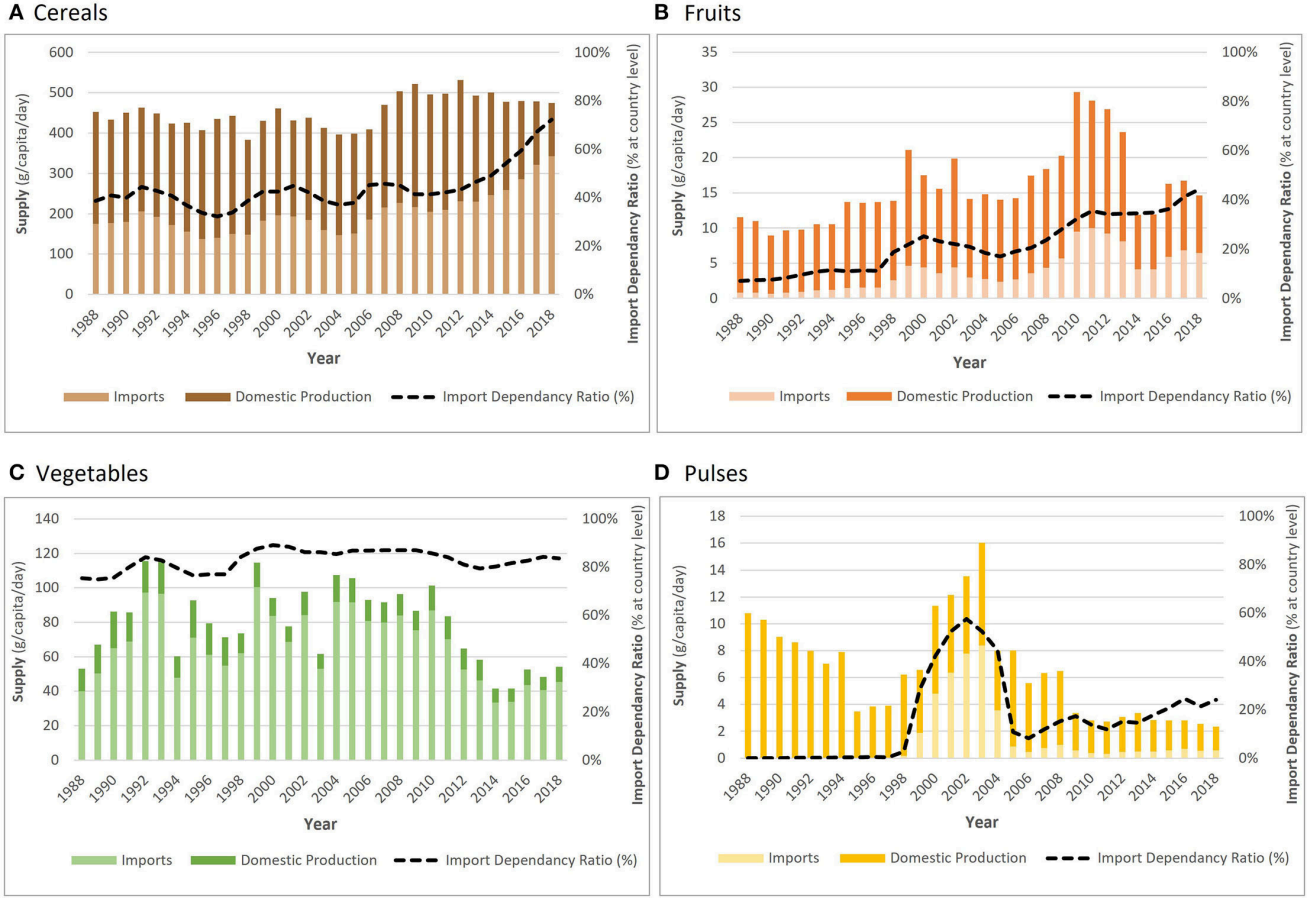
Changing total supply (g/capita/day) of **(A**) cereals, **(B)** fruits, **(C)** vegetables, and **(D)** pulses, split by imports (light) and domestic production (dark), between 1988 and 2018. Import dependency ratio (IDR) shown by black dashed line. Total supply uses the FAO food balance data, but the overlaid proportions of imports and exports, and hence IDRs, are 3-year rolling averages from the Kastner *et al* bilateral trade data (1987–2019).

Between 1988 and 2018, cereal supply in The Gambia increased by 4.9%, from 452.5 g per capita per day to 474.7 g per capita per day (Figure 1). Daily per capita fruit supply fluctuated, but saw an overall increase of 26.8%, from 11.5 to 14.6 g. The contribution of vegetables to total fruit and vegetable supply was consistently higher than for fruits, but overall supply only increased by 2.1%, from 53.0 g in 1988 to 54.2 g in 2018, despite peaking at 116 g in 1993. Daily per capita supply of pulses was very low, and decreased by 78.2%, from 10.8g to 2.4 g.

Increasing import dependency ratios (IDR) show that supply of all crop groups became more reliant on imports between 1988 and 2018. This was especially notable for cereals, where the IDR almost doubled (from 38.6 to 72.3%), and for fruits, where import reliance increased sixfold (IDR increased from 7.0 to 44.3%). Vegetable supply was the most reliant on imports (75.4% in 1988 and 83.7% in 2018), whereas pulses were mostly produced domestically.

### Vulnerability of The Gambia's Food System (1988–2018)

#### Climate Vulnerability of Supply

**Figure 2:**
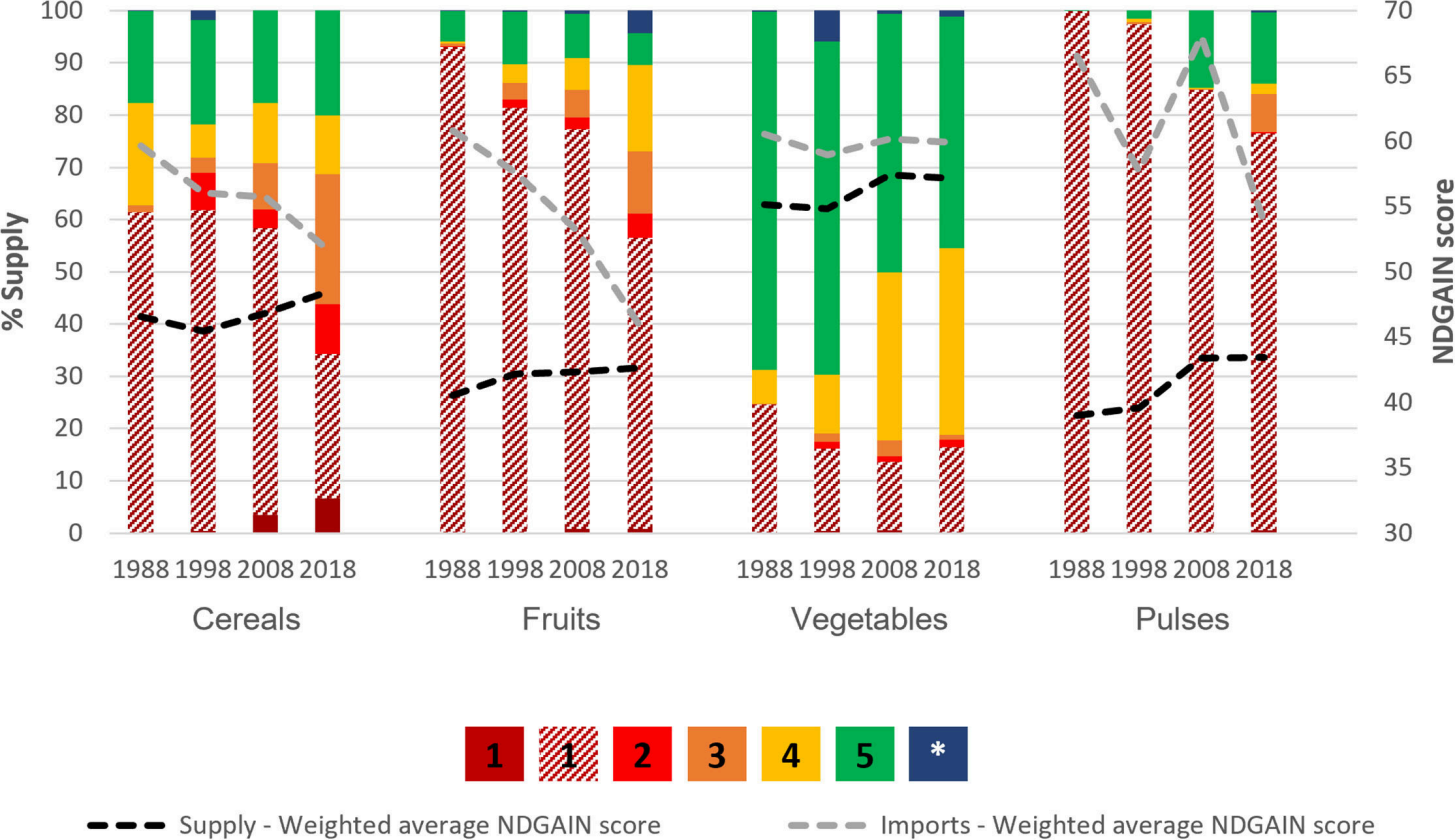
ND-GAIN vulnerability index of country of origin of cereals, fruits, vegetables and pulses supplied to The Gambia. The proportion of supply, within a given crop group and for a given year (1988, 1998, 2008 or 2018), originating in countries characterized by a given climate vulnerability status ([1–dark red] Extreme; [2–red] High; [3–orange] Intermediate to high; [4–yellow] Intermediate; [5–green] Low; or [*-blue] Unknown). Domestic production is shown with diagonal stripes and falls into the Extreme vulnerability (1) category, with the ND-GAIN country index score (2019) of The Gambia being 39.2. This figure also shows the weighted average ND-GAIN score of total supply (black dashed line) and of imports (gray dashed line). All data are 3-year rolling averages. All data between the years of 1988, and 2018 were analyzed, but we present 1988, 1998, 2008, and 2019 as evenly spaced time intervals for figure readability. Data for all years can be found in the supplementary materials (Supplementary Figure 4).

Not only have imports increased over time, but for cereals and fruits, more imports came from more climate-vulnerable countries. For example, in 1988, no cereals were being imported from extremely climate-vulnerable countries, but by 2018, this had increased to almost 10% (Figure 2). Generalized linear regressions show that per year, the import weighted-average ND-GAIN scores [0–100] deteriorated significantly [Cereals: −0.243 per year (95% CI: −0.278 to −0.208); Fruits: −0.374 per year (95% CI:-0.425 to −0.324)] (Table 1). By 2050, this would translate to almost 7 and 11 percentage points of decrease in ND-GAIN scores of cereal and fruit imports, respectively.

**Table 1:** Multilevel mixed-effects generalized linear model for weighted-average change in ND-GAIN score (climate vulnerability) of total supply and imports for cereals, fruits, vegetables, and pulses.

	**Total supply**		**Imports**	
**Food group**	**Average change in ND-GAIN score^**1**^ per year**	**95% CIs**	**Average change in ND-GAIN score^**1**^ per year**	**95% CIs**
Cereals	−0.007	−0.042, 0.028	−0.243	−0.268, −0.210**
Fruits	0.095	0.076, 0.114**	−0.374	−0.423, −0.326**
Vegetables	0.073	0.050, 0.960**	0.015	−0.016, 0.046
Pulses	0.138	0.052, 0.224*	−0.130	−0.269, 0.008

*p < 0.01, **p < 0.001; ^1^ 2019 ND-GAIN scores range from 28.3 to 76.8, whereby the lower the score, the more climate-vulnerable a country is considered.

The Gambia is classified as extremely vulnerable to climate change, and hence its domestic production falls into this category. Meeting supply needs by increasing reliance on imports has, so far, reduced the overall climate vulnerability of their supply for all crops except cereals, because most trade partners are less climate-vulnerable than The Gambia itself. Generalized linear regressions models revealed a statistically significant increase (indicating improvement) in the average ND-GAIN scores of total supply (imports plus domestic production minus exports) of fruits, vegetables and pulses (+0.095, +0.073 and +0.138, respectively, per year) (Table 1). Vegetables came from more climate stable countries than any of the other crops analyzed, with the majority supplied by imports from low or intermediately vulnerable countries (Figure 2).

**Figure 3:**
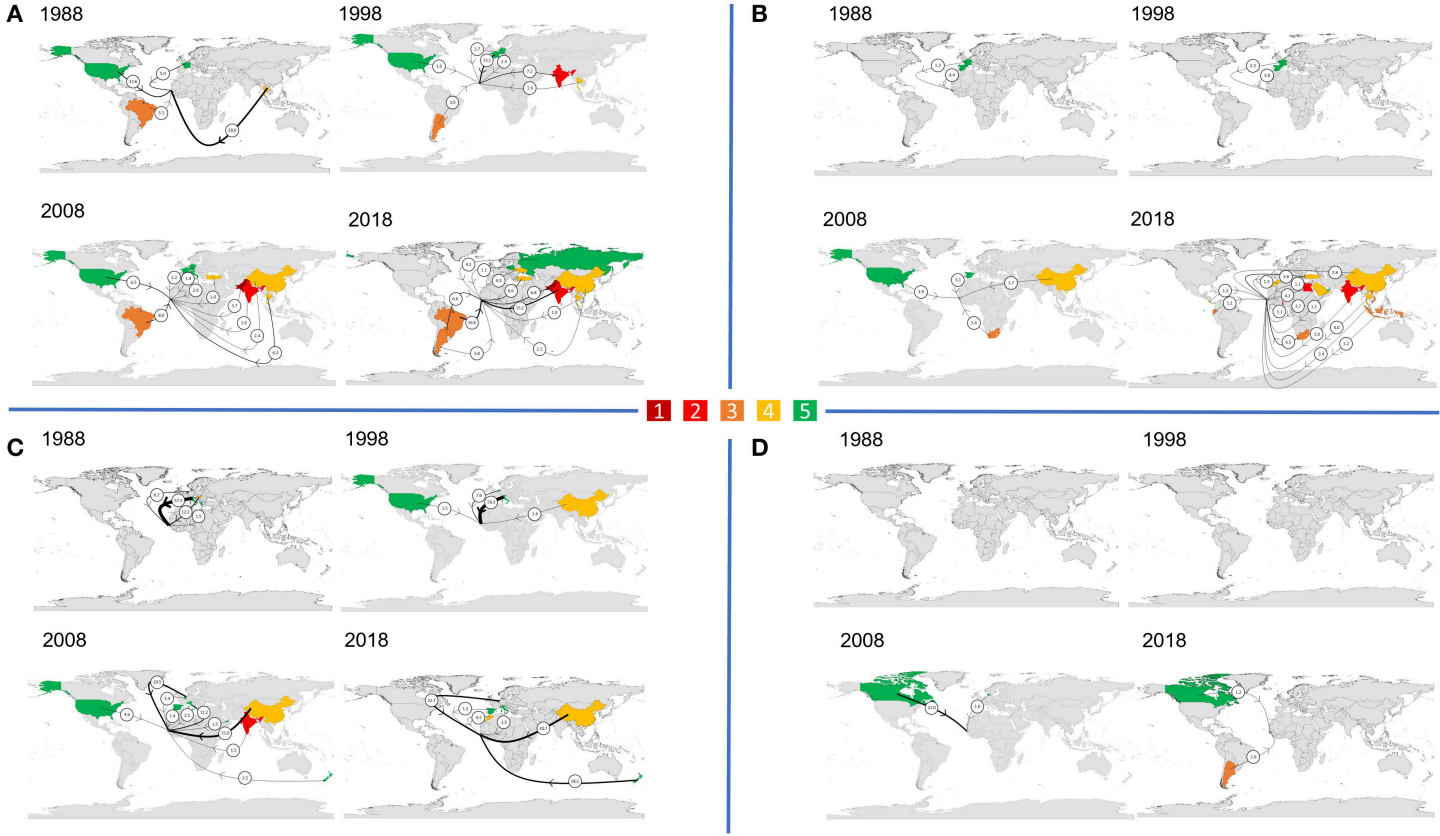
Climate vulnerability of trade partners (ND-GAIN index) contributing ≥1% of supply of **(A)** cereals **(B)** fruits **(C)** vegetables, **(D)** pulses, in 1988, 1998, 2008, and 2018. Amount the imported crop contributes to supply is represented by arrow thickness (not to scale) and numbers (%). There are five climate vulnerability categories: [1–dark red] Extreme; [2–red] High; [3–orange] Intermediate to high; [4–yellow] Intermediate; [5–green] Low.

Figure 3 shows the climate vulnerability of countries The Gambia relies on most for its supply of cereals, fruits, vegetables and pulses, in 1988, 1998, 2008, and 2018. For each crop group, the number of countries contributing at least 1% to supply (excluding The Gambia) has progressively increased between 1988 and 2018, and this is particularly notable for cereals (from 4 to 11 countries) and fruits (from 3 to 15 countries). In 1988, no highly or extremely climate-vulnerable countries were main cereal suppliers, but in 2018 The Gambia imported 15.2% from highly vulnerable India, and 6.8% from extremely vulnerable Pakistan. Where fruits are imported, the reliance on relatively climate stable Europe has reduced from 1988, and, by 2018, is much more global, with imports often having further to travel. Similarly for vegetables, climate-stable Italy has been replaced as the largest supplier by intermediately climate-vulnerable China. Having been solely domestically produced in 1988, by 2018 there are still only two other major suppliers of pulses, Argentina and Canada.

#### Water Stress of Supply

**Figure 4:**
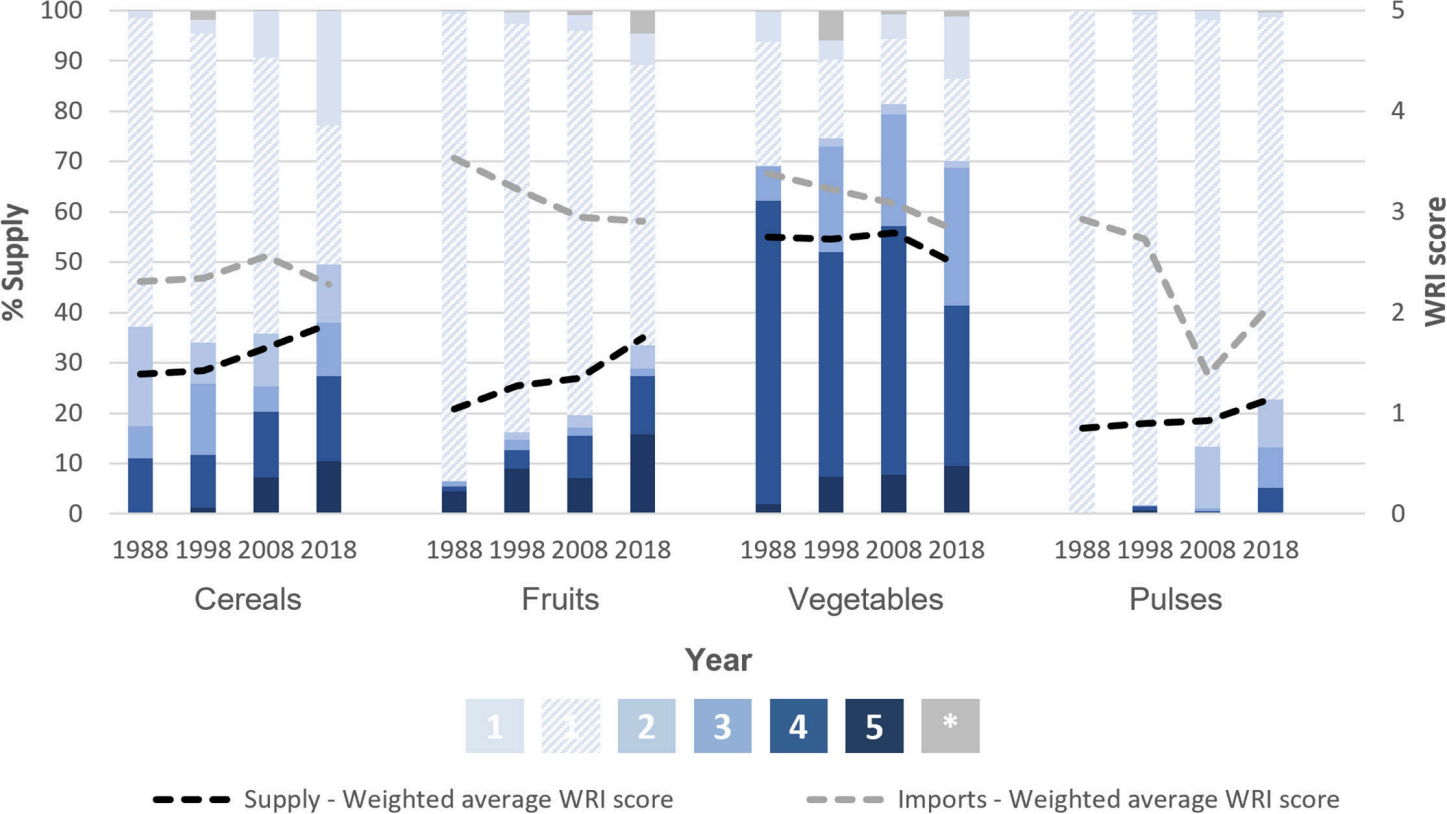
Projected water stress (WRI score) of country of origin of cereals, fruits, vegetables and pulses supplied to The Gambia. The proportion of supply, within a given crop group and for a given year (1988, 1998, 2008 or 2018), originating in countries of varying projected 2040 water stress levels (ratio of total water withdrawals to total renewable supply, WRI Aquaduct). There are five levels of water stress: [1] Low (<10%); [2] Low to medium (10–20%); [3] Medium to high (20–40%); [4] High (40–80%); [5] Extreme (>80%); plus those that are unknown [*-gray]. Projected water stress increases as colors move from light to dark blue. Domestic production is shown with diagonal stripes and falls into the low stress [1] category. This figure also shows the weighted average WRI score of total supply (black dashed line) and of imports (gray dashed line). All data are 3-year rolling averages. Analysis of 2040 projections based on a business-as-usual scenario. All data between the years of 1988 and 2018 were analyzed, but we present 1988, 1998, 2008, and 2019 as evenly spaced time intervals for figure readability. Data for all years can be found in the supplementary materials (Supplementary Figure 5).

The trends for projected water stress are somewhat the reversal of those for climate vulnerability. Comparing trade patterns in 1988 with those in 2018, total supply of both cereals and fruits became significantly more dependent on countries projected to be water stressed. Generalized linear models show a +0.015 (95% CI: 0.012 to 0.018) and +0.024 (95% CI: 0.020 to 0.027) yearly increase (indicating deterioration) in the WRI scores of cereal and fruit supplies, respectively. Since the WRI score of fruit imports actually significantly improved (−0.014 per year; 95% CI: −0.022 to −0.005), the more water stressed supply (0.024 per year; 95% CI:0.021 to 0.027) can be mostly explained by reduced domestic production, since the WRI categorize The Gambia as a country projected to experience only “low” water stress. For cereals, not only is there less domestic production, but the imports from countries projected to be water stressed have significantly increased, with an average yearly increase in WRI score of +0.013 (95% CI: 0.005 to 0.021) (Table 2). Figure 4 shows that the proportion of cereal supply imported from countries projected to be at least highly water stressed has more than doubled between 1988 and 2018 (11–27%).

**Table 2:** Multilevel mixed-effects generalized linear model for weighted-average change in WRI score (projected water stress in 2040) of total supply and imports for cereals, fruits, vegetables and pulses.

	**Total supply**		**Imports**	
**Food group**	**Average change in WRI score^**1**^ per year**	**95% CIs**	**Average change in WRI score^**1**^ per year**	**95% CIs**
Cereals	0.015	0.012, 0.018**	0.013	0.005, 0.021*
Fruits	0.024	0.021, 0.027**	−0.014	−0.022, −0.005*
Vegetables	−0.013	−0.019, −0.008**	−0.025	−0.030, −0.020**
Pulses	0.008	−0.005, 0.022	−0.041	−0.060, −0.023**

*p < 0.01, ^**^p < 0.001; ^1^ WRI scores range from 1 to 5, whereby the higher the score, the more water stressed a country is projected to be.

Compared to the other analyzed crops, a greater proportion of vegetable supply consistently came from countries projected to experience high or extremely high water stress. However, between 1988 and 2018 the proportion of supply from these countries decreased (60%–40%) (Figure 4), making it the only crop group where the average projected water stress of both total supply and imports has significantly reduced with time (Table 2).

**Figure 5:**
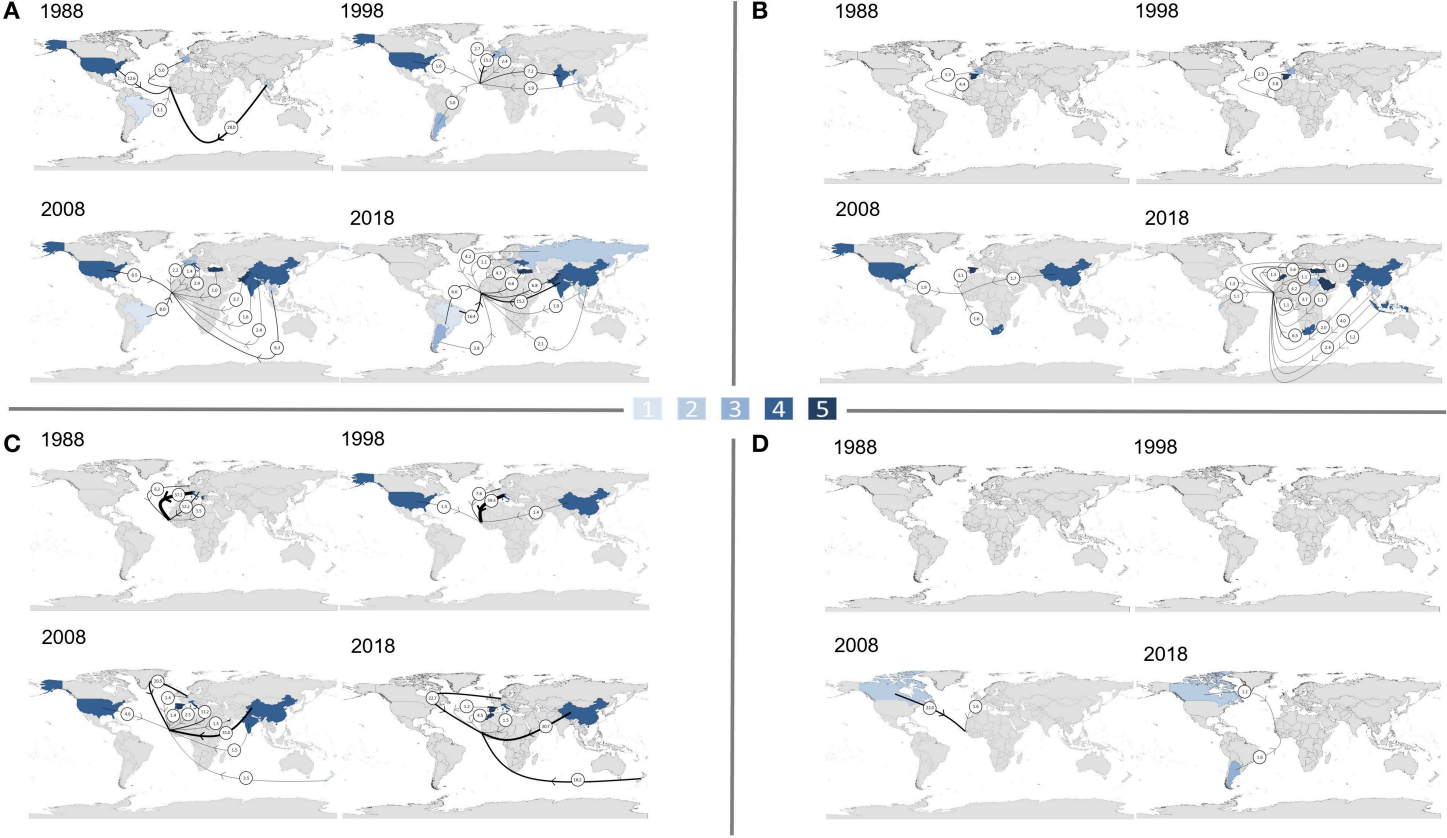
The projected water stress of trade partners (ratio of total water withdrawals to total renewable supply, WRI Aquaduct) contributing ≥1% of supply of **(A)** cereals **(B)** fruits **(C)** vegetables **(D)** pulses, in 1988, 1998, 2008, and 2018. Amount the imported crop contributes to supply is represented by arrow thickness (not to scale) and numbers (%). There are five water stress severity categories: [1] Low (<10%); [2] Low to medium (10–20%); [3] Medium to high (20–40%); [4] High (40–80%); [5] Extreme (>80%). The darker the color, the more water stressed a country is projected to be in 2040, in a business-as-usual scenario.

Figure 5 shows the projected water stress of countries The Gambia relied on most for its supply of cereals, fruits, vegetables and pulses, in 1988, 1998, 2008, and 2018. In 1988, The Gambia's largest trade partner for cereals was Thailand (28.0%), which is projected to experience low to medium water stress. This switched to Brazil in 2018, considered low water stress but only contributing 16.4%, with the next 15.2% supplied by highly water stressed India. The third and fourth largest cereal suppliers in 2018, Pakistan (6.8%) and Turkey (6.6%), are both projected to be extremely water stressed. Countries projected to be extremely water stressed also progressively featured among the top suppliers for fruits. In 1988, Spain (4.4%) was the only key trade partner projected to be extremely water stressed, but by 2018 there were four: Turkey (5.6%), Saudi Arabia (3.7%), Morocco (1.3%) and Lebanon (1.1%). A similar pattern can be seen for vegetable supply, whereby more countries that are projected to be water stressed featured as main suppliers; however, the replacement of vegetable supply from Italy (57.1%) with that from China (30.7%) and the Netherlands (22.7%) reduces the projected water stress of supply overall, because Italy is expected to be highly water stressed, while projected water stress in the Netherlands is only medium to high.

## Discussion

### Summary of Findings

Despite substantial fluctuations, between 1988 and 2018 The Gambia's per capita supply of cereals, fruits, and vegetables increased, but supply of pulses decreased. To achieve these increases in supply, reliance on imports has increased, and with it the number of major international trade partners. While this alleviates some of the pressure on The Gambia's domestic production, results on the implications for climate vulnerability and projected water stress are very mixed. Overall, supplies of all crops except cereals have become less reliant on climate-vulnerable countries between 1988 and 2018 (improved weighted-average ND-GAIN scores). However, vegetables were the only crop where supply hasn't become more reliant on countries projected to be water stressed by 2040 (deteriorating weighted-average WRI scores). To an extent, this is because The Gambia itself is classified as climate-vulnerable but not projected to become very water stressed, and therefore an increased IDR tends to improve the climate vulnerability of crop supplies but worsen their exposure to projected water stress. However, our analysis also shows that imports of fruits have progressively come from more climate-vulnerable countries, and imports of cereals have progressively come from both climate-vulnerable countries and those projected to be water stressed. Although vegetable supply is least reliant on climate-vulnerable countries, it is the most reliant on countries projected to experience water stress. However, it is the only analyzed crop where reliance on both climate-vulnerable countries and those projected to be water stressed has significantly reduced over time, improving the index scores of both supply and imports.

### Research in Context

Although there have been some notable improvements, a considerable proportion of The Gambia's supplies of cereals, fruits, vegetables and pulses are produced in countries vulnerable to environmental change. Therefore, while increases in agricultural productivity are expected to increase the resilience of many production areas, The Gambia's high dependence on imports may both increase and spread their risk of experiencing disruptions to supply.

The impact of environmental change on agricultural productivity will vary across crops and geographical areas (16, 29). For example, climate change may benefit some countries (primarily those in high latitudes) through increased crop yields, but most—and in particular tropical countries—will face declines (3, 4). The extent of these yield declines will vary, but they are expected to be greatest in Sub-Saharan Africa, where they will be three times the global average (16). Therefore, partly through shifts in comparative advantage, environmental change is likely to significantly change international trade patterns (16). As a net-importing tropical country, increasing reliance on countries that are climate-vulnerable and projected to be water-stressed could put The Gambia at a greater risk of negative productivity shocks and increased global food prices (16). Although a high climate vulnerability score is not necessarily directly linked to substantial reductions in yields or supply at the moment, the high risk profile related to climate change impacts, such as prolonged droughts, heat, extreme events and salinisation, make it more likely that these cascading risks could eventually reach a tipping point. Beyond such tipping points, yields and supplies would drastically drop, which could have far reaching consequences, and potentially lead to systemic failure in the production country (30). As an importer, The Gambia would likely be affected by these system failures as well, and hence high dependence on a number of highly climate change vulnerable countries would pose a substantial risk to the resilience of supply in the future.

The partially weather-mediated 2008 food crisis demonstrated that cereal productivity shocks can cause export bans and inflated prices, and hence poverty and malnutrition among the most vulnerable populations (31, 32). More recently, the COVID-19 pandemic has disrupted distribution chains and trade flows, causing an increase in world rice prices, with much uncertainty ahead (33). Low-income, import-dependant countries are expected to be most affected by reductions in exports and increases in price (34), and therefore The Gambia's increasing reliance on countries that are climate-vulnerable and projected to be water stressed to meet cereal demand raises some concern.

Results on fruits are more complex to interpret: while the increasing climate vulnerability gives reasons for concern, the reduced reliance on countries projected to be water stressed is positive. Given the already low supply of fruits and vegetables in The Gambia (35, 36), any vulnerabilities in supply risk further reductions in availability and affordability which would have public health implications. These could include micronutrient deficiencies and NCDs (6, 36, 37), both of which The Gambia need to address to minimize its triple burden of malnutrition as it progresses through the “Nutrition Transition”. Additionally, evidence suggests that globally, the poorest communities purchase and consume the least fruits and vegetables (38), and therefore any reductions in supply are likely to reduce consumption among these groups further, increasing health inequalities (39). According to the Global Burden of Disease Study, diets which are low in fruits and vegetables account for approximately a third of deaths and disability-adjusted life-years attributable to dietary risks in West Africa (40). More specifically, low fruit and vegetable intake is associated with the growing problem of obesity in The Gambia (41). Considering this nutritional context, the improvements in climate and water vulnerability of vegetable supply and imports are most welcomed.

### Policy Implications

#### Trade-Related Adaptations

Based on portfolio risk theory, increasing reliance on imports and maximizing the geographical diversity of supply could act as a mitigation strategy, increasing resilience by spreading the risk of regional supply disruption affecting The Gambia's imports (42). While this may mean many countries contributing only small amounts each to supply, this ensures a wide selection of trade partners where there are already established trading relationships and logistical arrangements. Should one country or region experience climate shocks and hence difficulty exporting produce, this may make it easier to urgently increase supply from others (depending on their capacity). Future policy action must balance these tradeoffs, among many others which fall outside the scope of this study, when considering food system adaptations to environmental change. Such considerations would include, but are by no means limited to, transport associated emissions where crops are being imported from further afield (43), being able to access other sources of imports in reasonable timeframes, and both the initial and ongoing costs of expanding the number of trade partners and hence potential impacts on affordability.

Within the context of very low availability of fruits, vegetables, and pulses, a substantial energy-dependency on cereals, (12, 44) and population growth that is occurring more rapidly in Sub-Saharan Africa than any other region in the world (45), there is an increasingly desperate need to scale up supply of these crops. As a result, despite The Gambia's high climate vulnerability, another option may include carefully managed increases in self-sufficiency. This would require consideration from an economic, environmental and risk perspective, given the complexity and tradeoffs of food systems transformation. For example, the implementation of the infrastructure might be economically feasible, but the potentially negative impact of increased competition for water withdrawals associated with increased crop production would need to be assessed.

Agricultural productivity across West Africa is currently low. The large gap between actual and potential yields of many staple crops suggests that there is great potential to increase future crop production in West Africa by increasing agricultural inputs (46). In addition, farming practices that respond to changes in temperature and precipitation can substantially reduce the negative impacts of climate change (11, 47). However, these adaptation strategies are very context-specific and need to be developed for individual fields and future climate conditions, which are highly uncertain. While it would require investment, research, and development, drought resistant crops and climate-smart agriculture could help mitigate some of the issues being faced because of predominantly rain-fed agriculture in The Gambia, and bolster the economy which is heavily reliant on agricultural productivity (48). Utilizing the potential adaptive capacity of The Gambia to scale up more environmentally stable domestic production, could be another strategy to increase their food system resilience to climate change.

#### Other Adaptations

There is no single pathway to resilience. Maximizing the climate resilience of The Gambia's food system will require a multisectoral approach, and, as such, other adaptations to be employed alongside altered trade. These may include consumptive policies, such as encouraging dietary substitutions with more climate-resilient crops like millets (49). This could be encouraged through initiatives promoting sustainable nutrition education, in conjunction with The Gambia's well-established school meals programme. These reach almost half of pre- and primary school children across all six regions of the country (50), and hence have the potential to shape more sustainable dietary habits in a large proportion of the future generation. Additionally, to support smallholders, the national school meals policy prioritizes a home-grown component (50), and therefore encouraging the inclusion of nutrient-rich and climate-resilient crops in this innovative approach would act at several levels of the food system.

Importantly, any adaptations must fit within the context of The Gambia's rapidly urbanizing population. Across West Africa, increases in population size and per capita incomes mean that urban food demand is expected to grow up to two to four times faster than rural demand. These factors also typically change dietary patterns, and therefore the expected increase in urban food demand is likely to be greater for relatively perishable commodities such as livestock products, fruits and vegetables, than for unprocessed starchy staples (51). Investments in reducing post-harvest loss, for example through tightly controlled cold chains, could help meet this demand by increasing the availability of fruits and vegetables (51). Additionally, policies to encourage small-scale subsistence farming and the development of home gardens may enhance food security among both rural and urban populations. They offer an opportunity to improve nutrition through increased local availability and food diversity, but go beyond this to improve livelihoods and promote entrepreneurship. Their success has been shown across Africa, as well as in other continents (52).

### Strengths and Limitations

By combining several large, comprehensive, open-source datasets, this study adds a unique contribution to the current literature on trade as a climate change adaptation strategy to increase food system resilience. While the results of this study are specific to The Gambia, the methods developed could be used for conducting similar analyses for almost any country in the world.

This study primarily relies on bilateral trade data from FAOSTAT. The quality of this data is somewhat dependent on information collected by the reporting country and may therefore vary between individual countries (53). It also relies on several assumptions and uses predictions to fill in missing data. This means that the trends identified in the study are likely to be accurate, but that the numbers themselves may be more indicative, varying from the true amounts. Additionally, issues with data accuracy are likely compounded by The Gambia's extensive informal cross-border trade, especially with Senegal (35). As such, official statistics may substantially under-estimate food trade and local supply. Indeed, a forthcoming analysis of The Gambia's 2015/16 Integrated Household Survey suggests that vegetable consumption could be at least double what is indicated by FAO (Supplementary Figure 2) (36). Regardless, availability of fruits and vegetables appears considerably less than the WHO's minimum daily consumption recommendation of 400 g, and FAOSTAT is the most comprehensive global database currently available, hence its wide use in peer-reviewed literature. Additionally, the reliability of the country of production data was increased by the running of origin-tracing algorithms, preventing the incorrect reporting of countries of last-layover as the countries of production (21). While we recognize important limitations in the data used, considering this analysis as a whole we therefore believe it to be the most reliable currently available.

Since the available trade data was quantified at a country-level, country-level estimates were also used for climate vulnerability and water stress. However, this approach is limited in that it does not capture intra-country variabilities, such as in countries with diverse eco-climatic zones and water resource conditions, and may therefore neglect local vulnerabilities. Similarly, seasonal variations are not accounted for. At present this is the best available data, but this limitation should be considered when interpreting results for potential policy action.

There are also limitations that are specific to the water stress metric. The WRI indicator is a ratio of the total withdrawals to total renewable supply in a given area. Although this is a widely applied metric (54), it gives a slightly misleading impression of water scarcity in The Gambia because it classifies the country as being projected to experience minimal water stress. A major reason for it falling into this category is the general absence of irrigation practices. However, this absence is also what makes the increasing frequency of extreme weather events—such as floods and droughts—the main risk associated with crop production in the country (12). Additionally, there is inherent uncertainty in estimating future conditions (including difficulty estimating the impact of technological improvement), and these figures are based on many assumptions (55).

Lastly, inferences about future outcomes that can be provided by a historical analysis such as this one are limited in the absence of policy or programmatic changes. The references to future pathways in the current study serve to alert policy makers and act as a starting point for considering a potential issue, but alternative analytical approaches and more comprehensive models with multiple inputs and controls are urgently needed to improve understanding and better inform decision making.

## Conclusion

We provide a novel insight into The Gambia's supply of cereals, fruits, vegetables and pulses. The very mixed results make it difficult to reach a single conclusion, but act to highlight the prevalence and extent of tradeoffs, and the important need for additional analysis of potential effects and policy actions. Despite some notable improvements in the environmental vulnerability of The Gambia's supply of nutritionally important crops (particularly vegetables), considerable, and in some cases increasing, proportions of their supply are produced in countries that are vulnerable to climate change and future water stress. This may have important implications for the future availability, affordability, and hence consumption of these crops in The Gambia, ultimately exacerbating the existing nutritional challenges.

Further research should investigate this for other nutritionally important crops that are expected to experience climate-mediated reductions in yields, such as nuts and seeds (4), and model the potential for small trade alterations to increase the resilience of The Gambia's supply of all crops. Most urgently, we need to prioritize models that explore and analyse the options to strengthen supply resilience together—such as altering trade patterns, ways of growing foods in an increasingly urbanized world, agricultural inputs and practices, and diets—as a combination of strategies is likely needed for maximum effect. A systems-level approach, with global coordination and continuing multisectoral efforts, is vital for ensuring an adequate supply of these nutritionally important crops, and hence safeguarding population nutritional health now and for generations to come.

## Data Availability Statement

The raw data supporting the conclusions of this article will be made available by the authors, without undue reservation.

## Author Contributions

GH and PS contributed to the conceptualization of the study and contributed to the formal analysis. GH and TK contributed to the data curation. RG and PS contributed to the funding acquisition. GH conducted the investigation. GH, ZA, TK, RG, AP, and PS designed the methodology. GH developed the visualization of results and wrote original darft. ZA, TC, AP, RG, and PS commented on/edited the draft. All authors contributed to the article and approved the submitted version.

## Funding

This study was funded by The Wellcome Trust Our Planet Our Heath Programme [Grant: 216021/Z/19/Z]. GH, ZA, TC, RG, and PS were supported under this grant. TK was supported by Deutsche Forschungsgemeinschaft (DFG, German Research Foundation) [project no. KA 4815/1-1]. AP was funded by the Medical Research Council (UK) and the Department for International Development (DFID) under the MRC/DFID Concordat agreement (MC-A760-5QX00). The funders had no role in the conceptualization, decision to publish or preparation of the manuscript.

## Conflict of Interest

The authors declare that the research was conducted in the absence of any commercial or financial relationships that could be construed as a potential conflict of interest.

## Publisher's Note

All claims expressed in this article are solely those of the authors and do not necessarily represent those of their affiliated organizations, or those of the publisher, the editors and the reviewers. Any product that may be evaluated in this article, or claim that may be made by its manufacturer, is not guaranteed or endorsed by the publisher.
