# Supplementary material for: Changes in Climate Vulnerability and Projected Water Stress of The Gambia's Food Supply Between 1988 and 2018: Trading With Trade-Offs
Source: Front Public Health. 2022 May 25;10:786071. doi: 10.3389/fpubh.2022.786071 (PMC9211751; doi:10.3389/fpubh.2022.786071)
Supplement: Supplementary file 1 [file Data_Sheet_1.zip › Figure S1.DOCX]

Supplementary Material

**SM Figure 1: Comparison of FAOSTAT food balance supply data and Global Dietary Database (GDD) dietary intake data between 1990 and 2018 for Cereals (brown), Fruits (orange), Vegetables (green) and Pulses (yellow).** Lighter shades of each colour represent FAO data, and darker shades represent GDD data. Left-hand y-axis refers to the daily per capita consumption of cereals and vegetables, and the right-hand y-axis refers to that of fruits and pulses
