# Supplementary material for: Changes in Climate Vulnerability and Projected Water Stress of The Gambia's Food Supply Between 1988 and 2018: Trading With Trade-Offs
Source: Front Public Health. 2022 May 25;10:786071. doi: 10.3389/fpubh.2022.786071 (PMC9211751; doi:10.3389/fpubh.2022.786071)
Supplement: Supplementary file 1 [file Data_Sheet_1.zip › Figure S2.DOCX]

Supplementary Material

**SM Figure 2: 2015 FAOSTAT Food Balance supply data vs 2015/16 Gambia Integrated Household Survey (IHS) dietary intake data vs 2015 Global Dietary Database (GDD) dietary intake data for relevant food groups.** FAOSTAT (blue), GDD (orange) and IHS (green)
