# Supplementary material for: Changes in Climate Vulnerability and Projected Water Stress of The Gambia's Food Supply Between 1988 and 2018: Trading With Trade-Offs
Source: Front Public Health. 2022 May 25;10:786071. doi: 10.3389/fpubh.2022.786071 (PMC9211751; doi:10.3389/fpubh.2022.786071)

Supplementary Material

**SM Figure 3:** **Change in crop supply (g/capita/day) and change in average climate vulnerability (NDGAIN score) of supply between 1988 and 2018.**


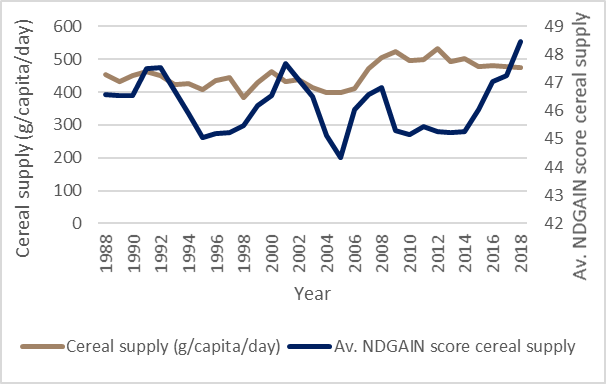

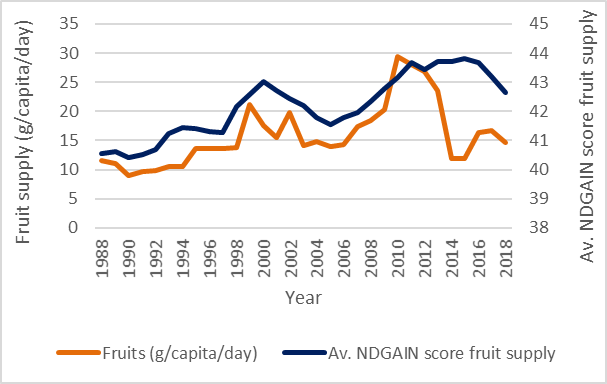

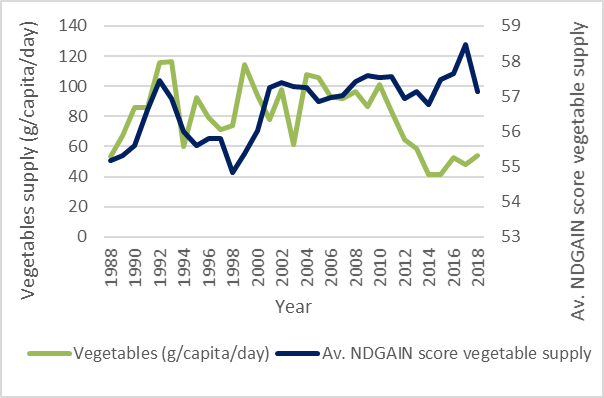

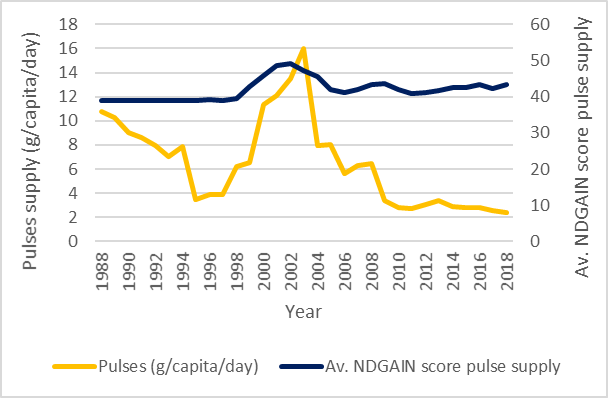

Supplement: Supplementary file 1 [file Data_Sheet_1.zip › Figure S3.DOCX]
