# Supplementary material for: Changes in Climate Vulnerability and Projected Water Stress of The Gambia's Food Supply Between 1988 and 2018: Trading With Trade-Offs
Source: Front Public Health. 2022 May 25;10:786071. doi: 10.3389/fpubh.2022.786071 (PMC9211751; doi:10.3389/fpubh.2022.786071)

Supplementary Material

**SM Figure 4:** **NDGAIN vulnerability index of country of origin of cereals, fruits, vegetables and pulses supplied to The Gambia.** The proportion of supply, within a given crop group across all years, originating in countries characterised by a given climate vulnerability status ([1 – dark red] Extreme; [2 - red] High; [3 - orange] Intermediate to high; [4 - yellow] Intermediate; [5 - green] Low; or [* - blue] Unknown). Domestic production is shown with diagonal stripes and falls into the Extreme vulnerability [1] category. Figure 4 also shows the weighted average NDGAIN score of total supply (black dashed line) and of imports (grey dashed line). All data are 3-year rolling averages.


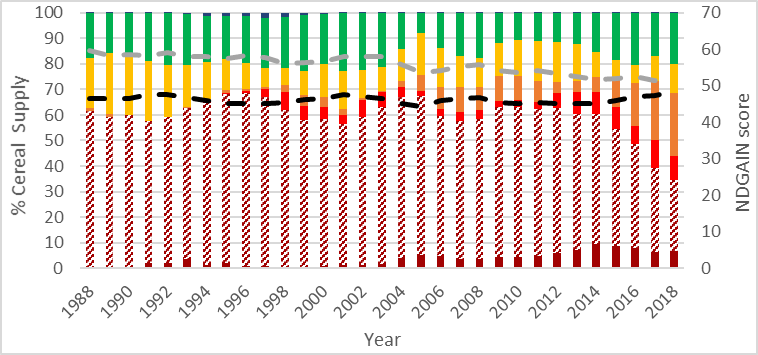

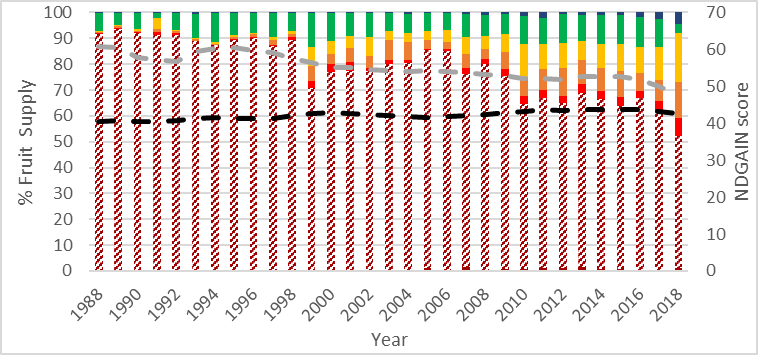

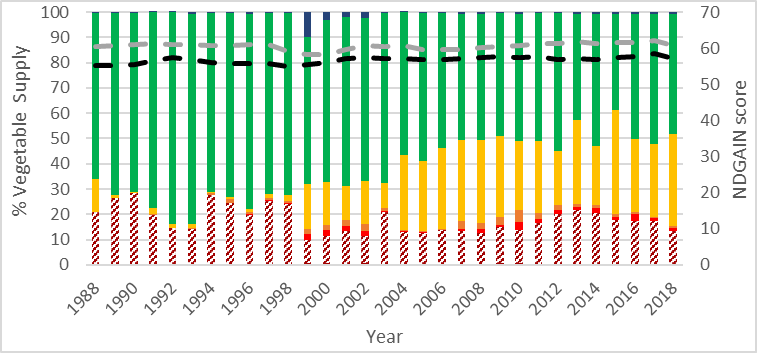

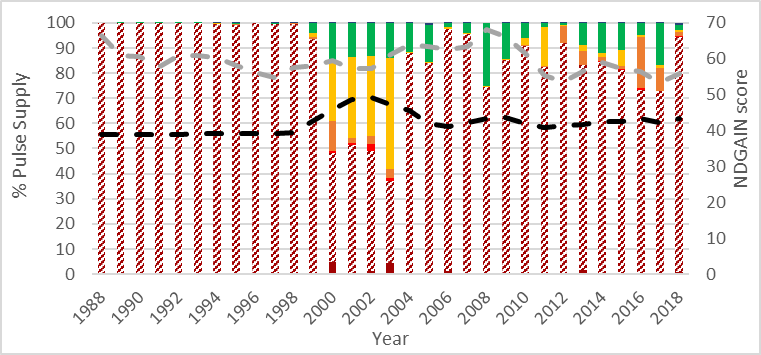

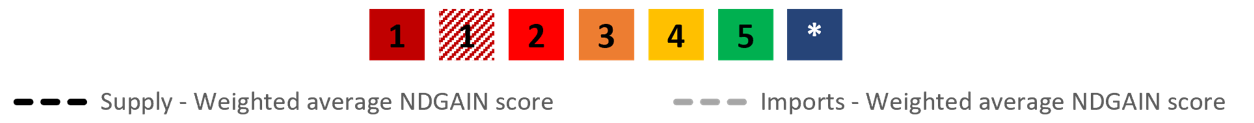

Supplement: Supplementary file 1 [file Data_Sheet_1.zip › Figure S4.DOCX]
