# Supplementary material for: Changes in Climate Vulnerability and Projected Water Stress of The Gambia's Food Supply Between 1988 and 2018: Trading With Trade-Offs
Source: Front Public Health. 2022 May 25;10:786071. doi: 10.3389/fpubh.2022.786071 (PMC9211751; doi:10.3389/fpubh.2022.786071)
Supplement: Supplementary file 1 [file Data_Sheet_1.zip › Figure S6.DOCX]

Supplementary Material

**SM Figure 6:** **Changing** **NDGAIN vulnerability index score of The Gambia (dashed red line), some of its most relied on trade partners for supply of cereals, fruits, vegetables and pulses (Brazil, China, France, India, Italy, The Netherlands, New Zealand and Thailand), as well as Norway and Chad (the countries with the highest and lowest NDGAIN country scores, respectively, between 1995 and 2019) to indicate the range of scores obtained by countries over time.**
