# Supplementary material for: Changes in Climate Vulnerability and Projected Water Stress of The Gambia's Food Supply Between 1988 and 2018: Trading With Trade-Offs
Source: Front Public Health. 2022 May 25;10:786071. doi: 10.3389/fpubh.2022.786071 (PMC9211751; doi:10.3389/fpubh.2022.786071)
Supplement: Supplementary file 1 [file Data_Sheet_1.zip › Table S1.DOCX]

Supplementary Material

# Supplementary Tables

**SM Table 1: Crops contributing to analysed food groups.**

| **Food Group** | **Crop** |
| --- | --- |
| Cereals | Barley, Buckwheat, Canary seed, Cereals (not otherwise specified), Fonio, Grain (mixed), Maize, Millet, Oats, Rice, Rye, Sorghum, Wheat |
| Fruits | Apples, Apricots, Bananas, Cherries, Currants, Dates, Figs, Fresh fruit (not otherwise specified), Tropical fresh fruit (not otherwise specified), Grapefruit (inc.Pomelos), Grapes, Kiwi fruits, Lemons and Limes, Mangoes, Mangosteens, Guavas, Oranges, Papayas, Peaches and Nectarines, Pears, Pineapples, Plantains, Plums and Sloes, Quinces, Strawberries, Tangerines, mandarins, Clementines, satsumas. |
| Vegetables | Artichokes, Asparagus, Aubergines, Beans (green), Cabbages and other brassicas, Carrots and Turnips, Cauliflowers and Broccoli, Chillies and Peppers (green), Cucumbers and Gherkins, Garlic, Leeks (and other alliaceous vegetables), Lettuce and Chicory, Maize (green), Mushrooms and Truffles, Onions (dry), Onions and Shallots (green), Peas (green), Pumpkins, Squash and Gourds, Spinach, Tomatoes, Vegetables (not otherwise specified). |
| Pulses | Beans (dry), Broad beans and horse beans (dry), Chickpeas, Lentils, Peas (dry), Pulses (not otherwise specified). |
