# Supplementary material for: Changes in Climate Vulnerability and Projected Water Stress of The Gambia's Food Supply Between 1988 and 2018: Trading With Trade-Offs
Source: Front Public Health. 2022 May 25;10:786071. doi: 10.3389/fpubh.2022.786071 (PMC9211751; doi:10.3389/fpubh.2022.786071)
Supplement: Supplementary file 1 [file Data_Sheet_1.zip › Table S2.DOCX]

Supplementary Material

**SM Table 2: Climate vulnerability of trade partners contributing ≥1% of supply of cereals, fruits, vegetables and pulses, in 1988, 1998, 2008 and 2018.** The proportion of supply, within a given crop group and for a given year (1988, 1998, 2008 or 2018), originating in countries characterised by a given climate vulnerability status (Extreme [dark red]; High [red]; Intermediate to high [orange]; Intermediate [yellow]; Low [green].

| **Cereals** | | | | | | | | **Fruits** | | | | | | | | **Vegetables** | | | | | | | | | | | **Pulses** | | | | | | | | | | |
| --- | --- | --- | --- | --- | --- | --- | --- | --- | --- | --- | --- | --- | --- | --- | --- | --- | --- | --- | --- | --- | --- | --- | --- | --- | --- | --- | --- | --- | --- | --- | --- | --- | --- | --- | --- | --- | --- |
| **1988** | | **1998** | | **2008** | | **2018** | | **1988** | | **1998** | | **2008** | | **2018** | | **1988** | | **1998** | | | **2008** | | | **2018** | | | **1988** | | | **1998** | | | **2008** | | | **2018** | |
| **Country** | **% Supply** | **Country** | **% Supply** | **Country** | **% Supply** | **Country** | **% Supply** | **Country** | **% Supply** | **Country** | **% Supply** | **Country** | **% Supply** | **Country** | **% Supply** | **Country** | **% Supply** | **Country** | **% Supply** | **Country** | | **% Supply** | **Country** | | **% Supply** | **Country** | | **% Supply** | **Country** | | **% Supply** | **Country** | | **% Supply** | **Country** | | **% Supply** |
| Gambia | 50.9 | Gambia | 64.2 | Gambia | 55.7 | Gambia | 26.2 | Gambia | 91.7 | Gambia | 89.4 | Gambia | 79.7 | Gambia | 51.0 | Italy | 57.1 | Italy | 59.3 | China | | 31.0 | China | | 30.7 | Gambia | | 100.0 | Gambia | | 99.6 | Gambia | | 74.5 | Gambia | | 93.4 |
| Thailand | 28.0 | France | 15.1 | Brazil | 8.0 | Brazil | 16.4 | Spain | 4.4 | Spain | 3.8 | Spain | 3.1 | South Africa | 6.5 | Gambia | 21.0 | Gambia | 24.1 | Netherlands | | 20.5 | Netherlands | | 22.7 |  | |  |  | |  | Canada | | 22.0 | Argentina | | 1.6 |
| United States of America | 12.6 | India | 7.2 | United States of America | 6.5 | India | 15.2 | France | 1.3 | France | 2.3 | United States of America | 1.9 | Turkey | 5.6 | Hungary | 12.2 | Netherlands | 7.6 | Gambia | | 12.2 | New Zealand | | 18.2 |  | |  |  | |  | Denmark | | 1.6 | Canada | | 1.2 |
| France | 5.0 | Argentina | 3.0 | Thailand | 6.3 | Pakistan | 6.8 |  |  |  |  | China | 1.7 | Egypt | 4.2 | Netherlands | 6.2 | United States of America | 1.5 | Italy | | 11.2 | Gambia | | 13.4 |  | |  |  | |  |  | |  |  | |  |
| Brazil | 3.1 | Belgium-Luxembourg | 2.7 | Pakistan | 3.7 | Turkey | 6.6 |  |  |  |  | South Africa | 1.6 | Thailand | 4.0 | Greece | 1.5 | China | 1.4 | United States of America | | 4.6 | Morocco | | 4.5 |  | |  |  | |  |  | |  |  | |  |
|  |  | Germany | 2.4 | Italy | 2.9 | Paraguay | 6.6 |  |  |  |  |  |  | Saudia Arabia | 3.7 |  |  |  |  | New Zealand | | 3.5 | Italy | | 1.5 |  | |  |  | |  |  | |  |  | |  |
|  |  | Thailand | 1.9 | China | 2.4 | Ukraine | 4.3 |  |  |  |  |  |  | China | 2.8 |  |  |  |  | Spain | | 2.5 | Spain | | 1.2 |  | |  |  | |  |  | |  |  | |  |
|  |  | United States of America | 1.6 | France | 2.2 | Russia | 4.2 |  |  |  |  |  |  | Vietnam | 2.4 |  |  |  |  | India | | 1.5 |  | |  |  | |  |  | |  |  | |  |  | |  |
|  |  |  |  | India | 1.6 | Argentina | 3.8 |  |  |  |  |  |  | India | 2.0 |  |  |  |  | UAE | | 1.5 |  | |  |  | |  |  | |  |  | |  |  | |  |
|  |  |  |  | Germany | 1.4 | China | 2.1 |  |  |  |  |  |  | Costa Rica | 1.3 |  |  |  |  | Portugal | | 1.4 |  | |  |  | |  |  | |  |  | |  |  | |  |
|  |  |  |  | Turkey | 1.0 | Thailand | 1.9 |  |  |  |  |  |  | Morocco | 1.3 |  |  |  |  | Belgium | | 1.4 |  | |  |  | |  |  | |  |  | |  |  | |  |
|  |  |  |  |  |  | Poland | 1.1 |  |  |  |  |  |  | Indonesia | 1.2 |  |  |  |  |  | |  |  | |  |  | |  |  | |  |  | |  |  | |  |
|  |  |  |  |  |  |  |  |  |  |  |  |  |  | UAE | 1.1 |  |  |  |  |  | |  |  | |  |  | |  |  | |  |  | |  |  | |  |
|  |  |  |  |  |  |  |  |  |  |  |  |  |  | Togo | 1.1 |  |  |  |  |  | |  |  | |  |  | |  |  | |  |  | |  |  | |  |
|  |  |  |  |  |  |  |  |  |  |  |  |  |  | Ecuador | 1.1 |  |  |  |  |  | |  |  | |  |  | |  |  | |  |  | |  |  | |  |
|  |  |  |  |  |  |  |  |  |  |  |  |  |  | Lebanon | 1.1 |  |  |  |  |  | |  |  | |  |  | |  |  | |  |  | |  |  | |  |
