# Supplementary material for: Changes in Climate Vulnerability and Projected Water Stress of The Gambia's Food Supply Between 1988 and 2018: Trading With Trade-Offs
Source: Front Public Health. 2022 May 25;10:786071. doi: 10.3389/fpubh.2022.786071 (PMC9211751; doi:10.3389/fpubh.2022.786071)
Supplement: Supplementary file 1 [file Data_Sheet_1.zip › Table S3.DOCX]

Supplementary Material

SM Table 3: Projected water stress of trade partners contributing ≥1% of supply of cereals, fruits, vegetables and pulses, in 1988, 1998, 2008 and 2018. The proportion of supply, within a given crop group and for a given year (1988, 1998, 2008 or 2018), originating in countries of varying projected 2040 water stress levels (ratio of total water withdrawals to total renewable supply, WRI Aquaduct). There are five levels of water stress: Low (<10%); Low to medium (10-20%); Medium to high (20-40%); High (40-80%); Extreme (>80%). The darker the colour, the more water stressed a country is projected to be in 2040, in a business-as-usual scenario.

| Cereals | | | | | | | | Fruits | | | | | | | | Vegetables | | | | | | | | Pulses | | | | | | | |
| --- | --- | --- | --- | --- | --- | --- | --- | --- | --- | --- | --- | --- | --- | --- | --- | --- | --- | --- | --- | --- | --- | --- | --- | --- | --- | --- | --- | --- | --- | --- | --- |
| 1988 | | 1998 | | 2008 | | 2018 | | 1988 | | 1998 | | 2008 | | 2018 | | 1988 | | 1998 | | 2008 | | 2018 | | 1988 | | 1998 | | 2008 | | 2018 | |
| Country | % Supply | Country | % Supply | Country | % Supply | Country | % Supply | Country | % Supply | Country | % Supply | Country | % Supply | Country | % Supply | Country | % Supply | Country | % Supply | Country | % Supply | Country | % Supply | Country | % Supply | Country | % Supply | Country | % Supply | Country | % Supply |
| Gambia | 50.9 | Gambia | 64.2 | Gambia | 55.7 | Gambia | 26.2 | Gambia | 91.7 | Gambia | 89.4 | Gambia | 79.7 | Gambia | 51.0 | Italy | 57.1 | Italy | 59.3 | China | 31.0 | China | 30.7 | Gambia | 100.0 | Gambia | 99.6 | Gambia | 74.5 | Gambia | 93.4 |
| Thailand | 28.0 | France | 15.1 | Brazil | 8.0 | Brazil | 16.4 | Spain | 4.4 | Spain | 3.8 | Spain | 3.1 | South Africa | 6.5 | Gambia | 21.0 | Gambia | 24.1 | Netherlands | 20.5 | Netherlands | 22.7 |  |  |  |  | Canada | 22.0 | Argentina | 1.6 |
| United States of America | 12.6 | India | 7.2 | United States of America | 6.5 | India | 15.2 | France | 1.3 | France | 2.3 | United States of America | 1.9 | Turkey | 5.6 | Hungary | 12.2 | Netherlands | 7.6 | Gambia | 12.2 | New Zealand | 18.2 |  |  |  |  | Denmark | 1.6 | Canada | 1.2 |
| France | 5.0 | Argentina | 3.0 | Thailand | 6.3 | Pakistan | 6.8 |  |  |  |  | China | 1.7 | Egypt | 4.2 | Netherlands | 6.2 | United States of America | 1.5 | Italy | 11.2 | Gambia | 13.4 |  |  |  |  |  |  |  |  |
| Brazil | 3.1 | Belgium-Luxembourg | 2.7 | Pakistan | 3.7 | Turkey | 6.6 |  |  |  |  | South Africa | 1.6 | Thailand | 4.0 | Greece | 1.5 | China | 1.4 | United States of America | 4.6 | Morocco | 4.5 |  |  |  |  |  |  |  |  |
|  |  | Germany | 2.4 | Italy | 2.9 | Paraguay | 6.6 |  |  |  |  |  |  | Saudia Arabia | 3.7 |  |  |  |  | New Zealand | 3.5 | Italy | 1.5 |  |  |  |  |  |  |  |  |
|  |  | Thailand | 1.9 | China | 2.4 | Ukraine | 4.3 |  |  |  |  |  |  | China | 2.8 |  |  |  |  | Spain | 2.5 | Spain | 1.2 |  |  |  |  |  |  |  |  |
|  |  | United States of America | 1.6 | France | 2.2 | Russia | 4.2 |  |  |  |  |  |  | Vietnam | 2.4 |  |  |  |  | India | 1.5 |  |  |  |  |  |  |  |  |  |  |
|  |  |  |  | India | 1.6 | Argentina | 3.8 |  |  |  |  |  |  | India | 2.0 |  |  |  |  | UAE | 1.5 |  |  |  |  |  |  |  |  |  |  |
|  |  |  |  | Germany | 1.4 | China | 2.1 |  |  |  |  |  |  | Costa Rica | 1.3 |  |  |  |  | Portugal | 1.4 |  |  |  |  |  |  |  |  |  |  |
|  |  |  |  | Turkey | 1.0 | Thailand | 1.9 |  |  |  |  |  |  | Morocco | 1.3 |  |  |  |  | Belgium | 1.4 |  |  |  |  |  |  |  |  |  |  |
|  |  |  |  |  |  | Poland | 1.1 |  |  |  |  |  |  | Indonesia | 1.2 |  |  |  |  |  |  |  |  |  |  |  |  |  |  |  |  |
|  |  |  |  |  |  |  |  |  |  |  |  |  |  | UAE | 1.1 |  |  |  |  |  |  |  |  |  |  |  |  |  |  |  |  |
|  |  |  |  |  |  |  |  |  |  |  |  |  |  | Togo | 1.1 |  |  |  |  |  |  |  |  |  |  |  |  |  |  |  |  |
|  |  |  |  |  |  |  |  |  |  |  |  |  |  | Ecuador | 1.1 |  |  |  |  |  |  |  |  |  |  |  |  |  |  |  |  |
|  |  |  |  |  |  |  |  |  |  |  |  |  |  | Lebanon | 1.1 |  |  |  |  |  |  |  |  |  |  |  |  |  |  |  |  |
